# Supplementary material for: Cycles in seizure duration and their underlying dynamics in the tetanus toxin rat model
Source: Brain Commun. 2025 Sep 18;7(5):fcaf364. doi: 10.1093/braincomms/fcaf364 (PMC12492870; doi:10.1093/braincomms/fcaf364)
Supplement: fcaf364_Supplementary_Data [file fcaf364_supplementary_data.docx]

# Supplementary material


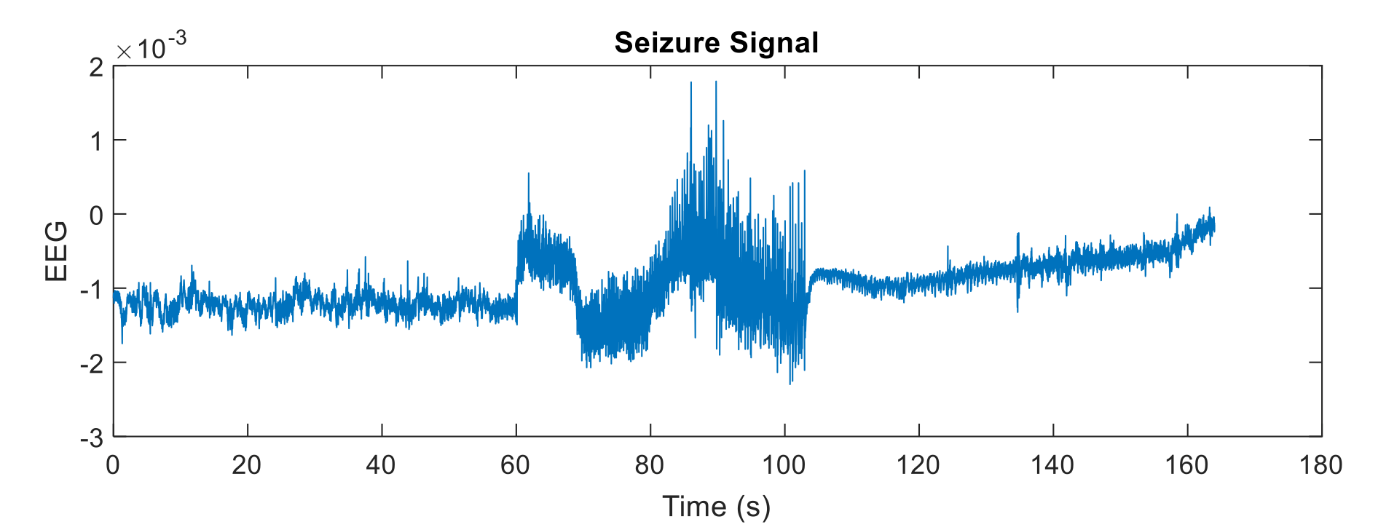


Supplementary Figure 1: Representative EEG trace from the epilepsy model group, demonstrating the presence of a spontaneous seizure event.


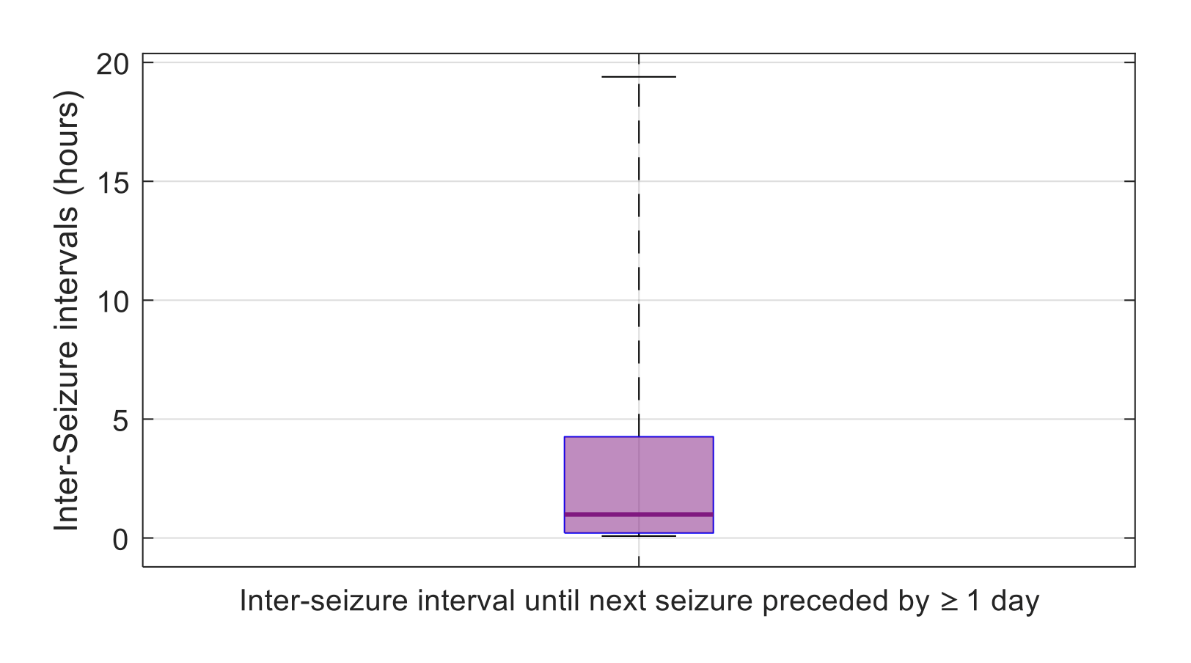


Supplementary Figure 2: Inter-seizure intervals following seizures that were preceded by ≥1 day of seizure free time. The majority of these subsequent seizures occurred within a few hours, suggesting that seizures following long intervals are frequently followed by shorter-than-average recurrence times.
